# Suppression of hypoxia-induced excessive angiogenesis by metformin via elevating tumor blood perfusion

**DOI:** 10.18632/oncotarget.18029

**Published:** 2017-05-19

**Authors:** Ji-Chang Wang, Guang-Yue Li, Ping-Ping Li, Xin Sun, Wei-Ming Li, Yan Li, Shao-Ying Lu, Pei-Jun Liu

**Affiliations:** ^1^ Department of Vascular Surgery, First Affiliated Hospital of Xi’an Jiaotong University, Xi’an, Shaanxi Province, 710061, P.R.China; ^2^ Center for Translational Medicine, First Affiliated Hospital of Xi’an Jiaotong University, Xi’an, Shaanxi Province, 710061, P.R.China; ^3^ Department of Science and Technology, First Affiliated Hospital of Xi’an Jiaotong University, Xi’an, Shaanxi Province, 710061, P.R.China; ^4^ Department of Thoracic Surgery and Oncology, First Affiliated Hospital of Xi’an Jiaotong University, Xi’an, Shaanxi Province, 710061, P.R.China

**Keywords:** metformin, inhibition of tumor angiogenesis, hypoxia, elevating blood perfusion, HIF-1α

## Abstract

The anti-diabetic metformin has been demonstrated to be effective in suppression of tumor progression via multiple mechanisms, in which angiogenic inhibition is involved. Hypoxia is a common feather of malignant tumor and promotes angiogenesis via induction of pro-angiogenic factors. However, the effect of metformin on tumor hypoxia and the association with angiogenic inhibition are still unclear. In the current study, we investigated the effects of metformin on both tumor blood perfusion and hypoxia-induced excessive angiogenesis. In the tumor region adjacent to necrosis, aberrantly excessive angiogenesis resulted from hypoperfusion-induced intense hypoxia and greatly contributed to the high average levels of both microvessel density and vascular branch density. Metformin administration increased the percentage of lectin-perfused vessels and reduced hypoxyprobe-positive area. This metformin-induced amelioration of hypoxia was accompanied by a significant reduction in expressions of both HIF-1α and angiogenesis-associated factors (AAFs). Consequently, inhibited excessive angiogenesis in hypoxic peri-necrotic region was observed in metformin-treated tumor. Further stable knockdown of HIF-1α abrogated hypoxia-induced AAFs *in vitro* and reduced both microvessel density and area of fitc-conjugated dextran that leaked outside the vascular lumen. Taken together, metformin ameliorated tumor hypoxia and restrained HIF-1α-induced expressions of AAFs through elevating tumor blood perfusion, thus suppressing the excessive tumor angiogenesis.

## INTRODUCTION

Malignant tumor is one of the disorders characterized by abnormal or excessive angiogenesis [1], which has been deeply implicated in cancer progression. When dysregulated, aberrantly formed tumor vessels by angiogenesis are always tortuous, dilated and excessively branched [2]. Consequently, this disorganized vasculature is inefficient for blood supply and lead to a hypoxic tumor microenvironment that has a fundamental role for cancer progression [3]. Suppression of tumor angiogenesis by molecular inhibitors has provided new opportunities for cancer management and greatly broadened our understanding of the role of angiogenesis in tumor progression. However, current anti-angiogenesis treatments, in which vascular endothelial growth factor (VEGF) inhibitors are key components, have been greatly challenged for therapeutic resistance [4] and induction of metastasis [5]. Thus, from drug discovery standpoint, a promising solution in overcoming this challenge is to explore new drug or new aspects of old drugs.

Epidemiological and experimental studies have revealed that the anti-diabetic metformin was able to reduce risk of various types of cancer [6] and inhibit tumor progression [7]. In the past few decades, metformin was extensively applied for the management of diabetes and its good tolerance has been deeply verified in the long-term clinical use. Due to its clinical safety and anti-tumor activities demonstrated by pre-clinical researches [8, 9], researchers are increasingly concerned to understand its anti-tumor potentials. Recently published articles have expanded this mechanistic profile by providing the novel insight into angiogenic suppression [10, 11]. However, the mechanism underlying metformin-induced angiogenesis inhibition remains an enigma.

In this paper, efforts were devoted to investigate the effects of metformin on hypoxia-induced angiogenesis for various reasons. First, metformin has been shown to inhibit hypoxia inducible factor 1 alpha (HIF-1α)-VEGF signaling, but the association with HIF-1α-induced other AAFs than VEGF is still undefined. Second, hypoxia induces angiogenesis by stimulating the secretion of angiogenesis-associated factors (AAFs) [12]. However, it is unclear whether metformin inhibits angiogenesis by ameliorating tumor hypoxia. Third, tumor starts to experience hypoxia when size reach 2 mm^2^ and hypoxia induces HIF-1α accumulation. Although metformin had been shown to increase tumor oxygenation [13], it remains incompletely understood about whether metformin inhibits HIF-1α-induced angiogenesis by increasing tumor blood perfusion.

In the present study, we investigated the effects of metformin on inducing anti-angiogenesis in different tumor regions, especially in peri-necrotic region (PNR) that has been demonstrated to be hypoxic. We used 4T1 murine breast cancer cell, which would encounter hypoxia and necrosis *in vivo* [14], to investigate if metformin could be used for inhibition of hypoxia-associated angiogenesis. Our results of both *in vivo* and *in vitro* experiments showed that metformin restrained hypoxia-induced excessive angiogenesis in PNR by suppressing HIF-1α-induced pro-angiogenic factors via elevating tumor blood perfusion.

## RESULTS

### Excessive angiogenesis occurred in tumor region adjacent to necrosis

**Figure 1:**
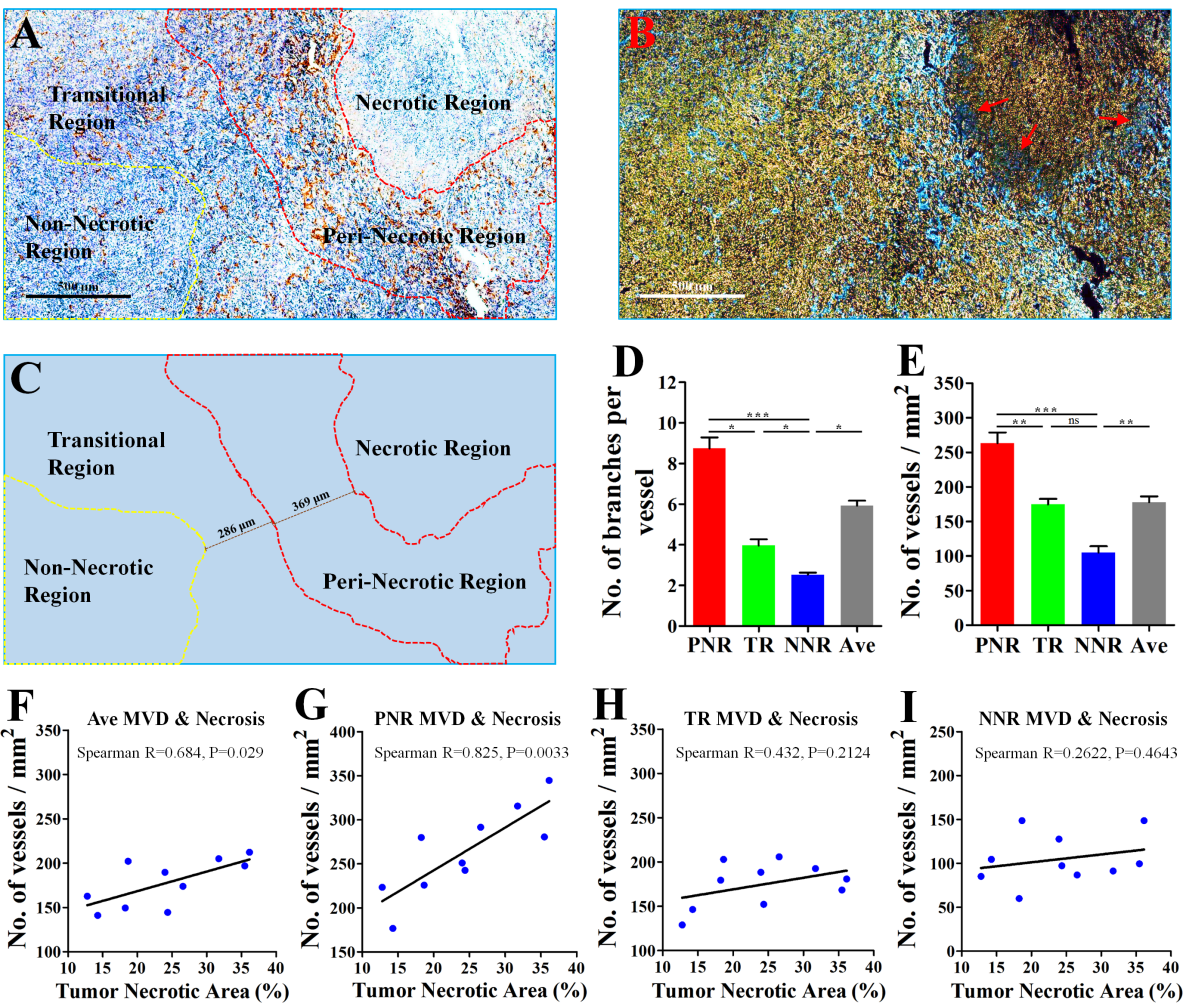
Excessive angiogenesis occurred in the peri-necrotic tumor region 4T1 breast cancer cells were orthotopically inoculated into the fat pad of 4th breast of BALB/c mice. 28 days later, the whole tumor tissue from untreated mouse was extracted for further analysis. (**A**) Representative image (Immunological Histological Chemistry) for showing CD31^+^ vessels distributed in peri-necrotic region, transitional region and the non-necrotic region. Scale bar: 500 μm. (**B**) The color-reverse image and (**C**) regional distribution map of panel (A) for revealing the discrepancy of angiogenic ability of vessels distributed in different tumor regions. In the area adjacent to tumor necrosis, vessels exhibited the abnormally high abilities of sprouting and branching. Red tri-angle indicates vessels with decreased CD31 expression in the edge region of tumor necrosis. Scale bar: 500 μm. The peri-necrotic and non-necrotic regions were surrounded by red and yellow dotted lines, respectively. Quantification of (**D**) microvessel density (MVD, No. of vessels per mm^2^) and (**E**) vascular branch density (No. of vascular branches per vessel) of regions having different distance from tumor necrosis (*n* = 10). Spearman analysis for evaluation of the linear correlation between percentage of tumor necrotic area and MVD of (**F**) the whole tumor area, (**G**) peri-necrotic region (PNR), (**H**) transitional region (TR) and (**I**) non-necrotic region (NNR). Quantitative data are indicated as mean ± SEM. *p < 0.05; **p < 0.01; ***p < 0.001; ns indicates no significant (*P* > 0.05).

Derived from a mouse mammary gland cancer [16], 4T1 cell line could be orthotopically transplanted into the BALB/c mouse [14], which has a normal immune system. According to different angiogenic abilities of vessels (Figure 1A–1C), 4T1 tumor was divided into peri-necrotic region (PNR), transitional region (TR) and non-necrotic region (NNR). IHC staining for CD31, a specific endothelial marker, showed that tumor region adjacent to necrosis exhibited an aberrantly high microvessel density (MVD) and vascular branch density. Decreased CD31 expression of tumor vessels was found in the edge region of necrosis (Red arrows; Figure 1B). Critically, vessels in PNR and TR had higher vascular abilities of both sprouting and branching than those in NNR (Figure 1A–1E). These results indicate that the closer the vessel lies to necrosis, the more excessive angiogenic phenotype is likely to be observed.

### MVD in PNR was linearly correlated with necrotic area

PNR had a higher MVD than average MVD of the whole tumor, while NNR had a lower MVD (Figure 1E), suggesting excessive angiogenesis in PNR contributed to high MVD of the whole tumor. Since hypoxia induces tissue necrosis [17], we next investigated whether tumor necrosis was correlated with excessive angiogenesis in peri-necrotic region. Results of spearman linear correlation analysis showed (Figure 1F–1I) that both average MVD and PNR MVD had significant linear correlation with percentage of necrotic region in untreated 4T1 cancer. Critically, PNR MVD exhibited a higher statistical significance than average MVD. However, MVD of both NNR and TR had no linear correlation with percentage of necrotic region. Spearman R for PNR MVD was 0.825 (*P* = 0.0033); spearman R for average MVD was (*P* = 0.029); spearman R for TR MVD was 0.432 (*P* = 0.2124); spearman R for NNR MVD was 0.2622 (*P* = 0.4643). These data indicated that correlation between average MVD and tumor necrosis resulted from PNR MVD but not MVD of TR and NNR. This speculation was further supported by the fact that only the correlation between PNR MVD and average MVD was statistically significant (Supplementary Figure 1A–1C).

### Hypoperfusion resulted in hypoxia-induced excessive angiogenesis in PNR

**Figure 2:**
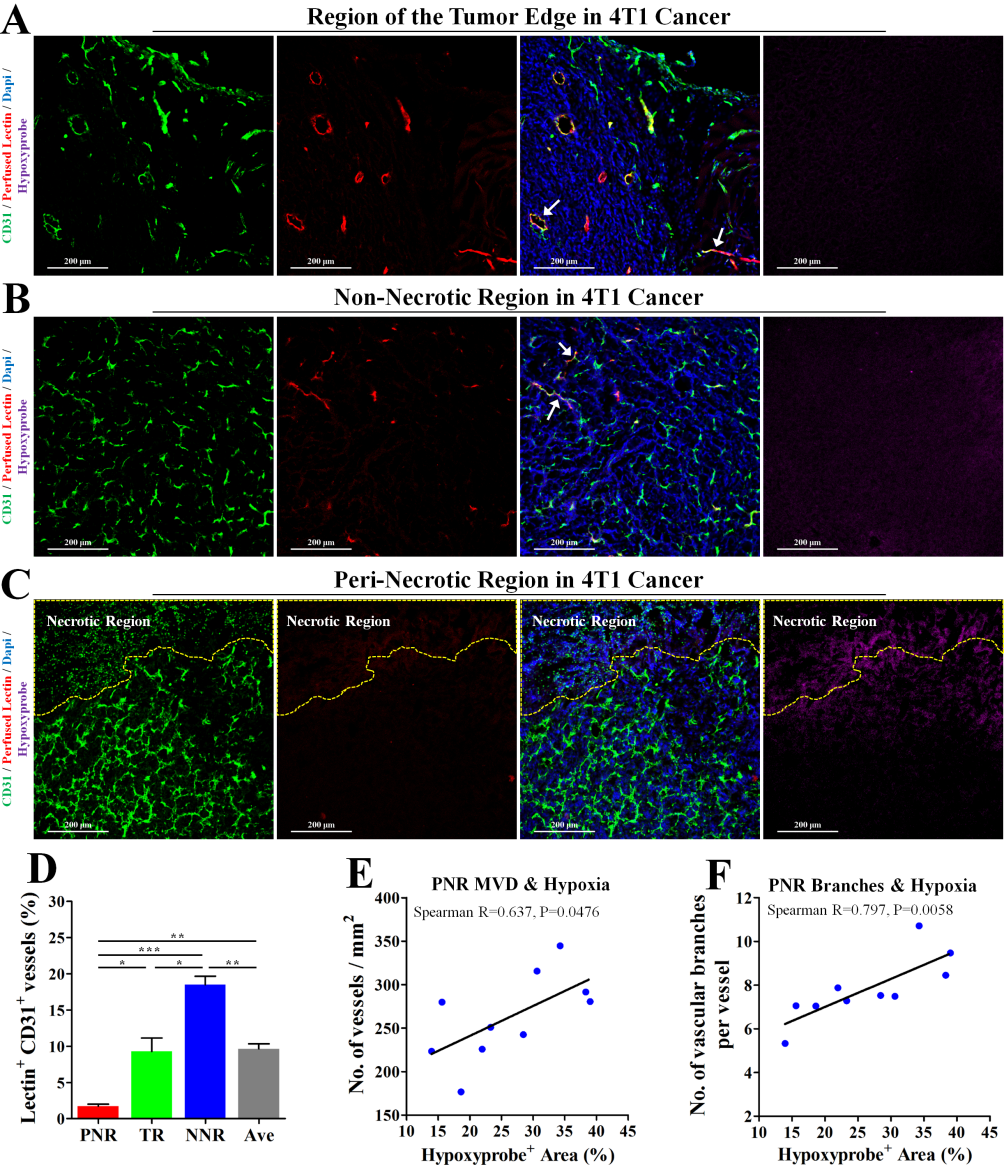
Hypoperfusion resulted in tumor necrosis and hypoxia-associated excessive angiogenesis in peri-necrotic region The wholly extracted 4T1 tumors were fixed by 4% paraformaldehyde and cut into 6μm thick frozen section for further immunofluorescent staining. 2 h and 15 min before extraction, pimonidazole hydrochloride and TRITC-conjugated lectin was injected into mouse tail vein, respectively. Staining for CD31 (Green), TRITC-conjugated lectin (Red) and pimonidazole hydrochloride (Violet) in (**A**) tumor edge, (**B**) non-necrotic and (**C**) peri-necrotic regions of 4T1 tumors from untreated BALB/c mouse. The necrotic region was surrounded by yellow dotted line. Scale bar: 200 μm. (**D**) Percentage of perfused-lectin^+^ CD31^+^ vessels in all CD31^+^ vessels of the whole tumor area (Ave indicates average perfusion level), peri-necrotic region (PNR), transitional region (TR) and non-necrotic region (NNR). *n* = 10. (**E** and **F**) Spearman analysis for evaluation of the linear correlation between percentage of hypoxic area (detected by IHC staining for hypoxyprobe) and (E) MVD or (F) vascular branch density in peri-necrotic region (PNR). Quantitative data are indicated as mean ± SEM. *p < 0.05; **p < 0.01; ***p < 0.001; ns indicates no significant (*P* > 0.05).

To investigate the relationship between perfusion status and hypoxia-associated angiogenesis, 4T1 tumor bearing-mice were intravenously injected with TRITC-conjugated lectin and PIMO (for hypoxia detection). Intravenously injected lectin is able to bind to endothelial cell (EC), thus labeling the blood-perfused vascular endothelial layer. Further immunofluorescent staining (Figure 2A, 2B) showed that vessels in both tumor edge and non-necrotic regions could be perfused by lectin-contained blood, indicating a good blood perfusion status in both regions. However, only 1.72% vessels were labelled by intravenously injected lectin in the peri-necrotic region (Figure 2C, 2D), where severe hypoxia was also found (indicated by positive hypoxyprobe signal). Furthermore, percentage of hypoxic tumor area had significantly linear correlation with both MVD and vascular branch density in PNR (Figure 2E, 2F). Spearman R for PNR MVD was 0.637 (*P* = 0.0476); spearman R for PNR vascular branch density was 0.797 (*P* = 0.0058). These data demonstrate hypoperfusion-induced hypoxia leads to excessive angiogenesis.

### Metformin reduced necrosis, inhibited angiogenesis and promoted vascular pericyte coverage

**Figure 3:**
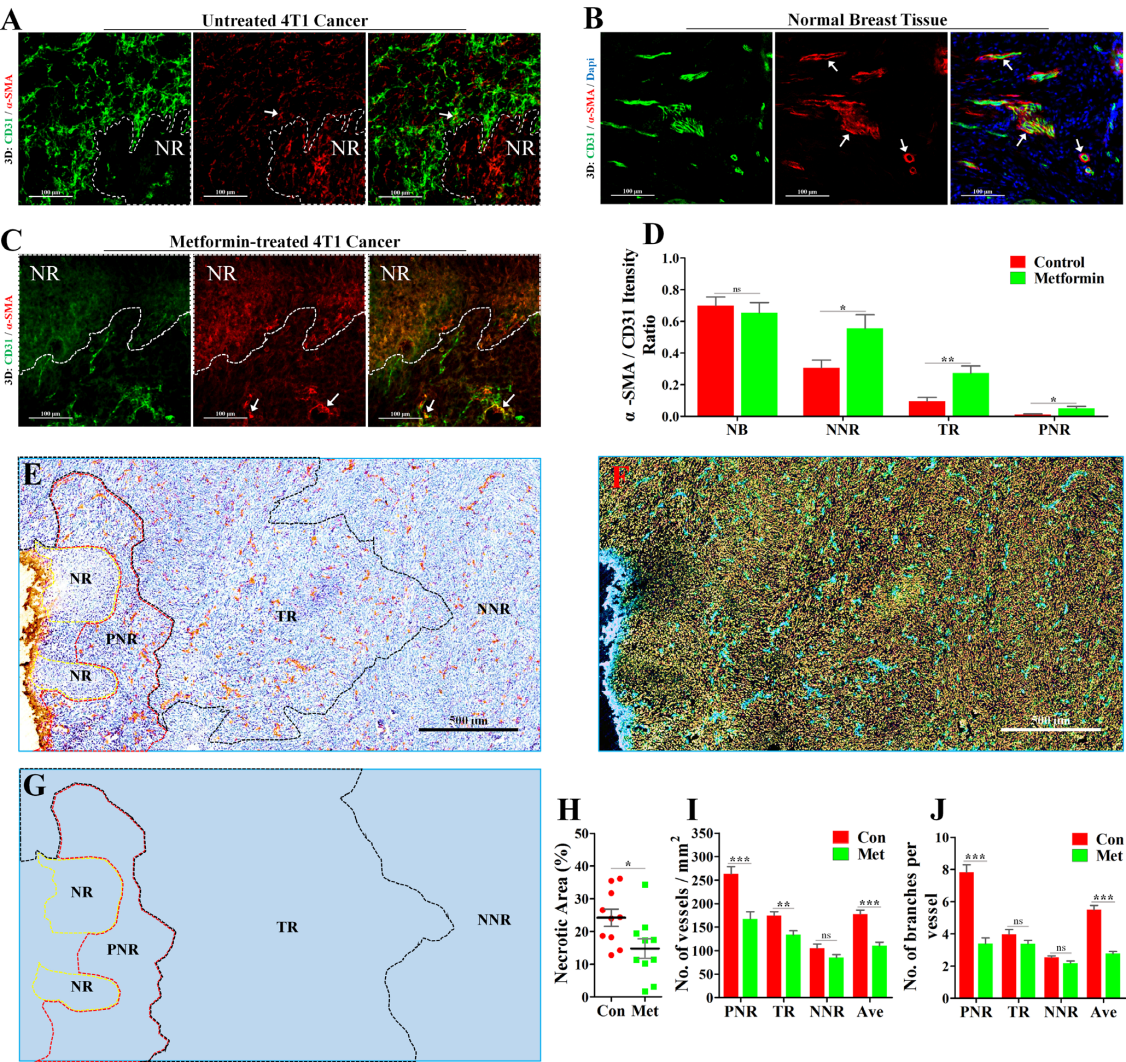
Increase of vascular maturity contributed to metformin-induced reduction of necrosis and MVD 4T1 tumor-bearing BALB/c mice were orally treated with metformin (1.5 mg/mL) for 21 days before extraction. The wholly extracted 4T1 tumors were fixed by 4% paraformaldehyde and cut into 40μm thick frozen section for further 3D-reconstruction of immunofluorescent signal. 3D-reconstruction of fluorescent signal of CD31 (Green) and α-SMA (indicating vascular smooth muscle cell, VSMCs) in (**A**) untreated 4T1 tumor (Con), (**B**) normal breatst tissue and (**C**) metformin-treated 4T1 tumor (Met). α-SMA signal co-located with CD31 signal indicate VSMCs that covered the tumor vascular lumen. (**D**) Quantification of ratio of co-located fluorescent α-SMA to CD31 in NB (normal breast), PNR (peri-necrotic region), TR (transitional region) and NNR (non-necrotic region). Scale bar: 100 μm. (**E**) Representative image (Immunological Histological Chemistry) for showing CD31^+^ vessels distributed in peri-necrotic region, transitional region and non-necrotic region of metformin-treated 4T1 tumor. Scale bar: 500 μm. (**F**) The color-reverse image and (**G**) regional distribution map of panel (E) for revealing angiogenic ability of vessels distributed in different regions of metformin-treated tumor. Scale bar: 500μm. The peri-necrotic and non-necrotic regions were surrounded by red and yellow dotted lines, respectively. (**H**) Quantification of percentage of necrotic area in 4T1 tumors from untreated or metformin-treated BALB/c mice (*n* = 10). Quantification of (**I**) microvessel density (MVD, No. of vessels per mm^2^) and (**J**) vascular branch density (No. of vascular branches per vessel) of different regions in 4T1 tumors from untreated or metformin-treated BALB/c mice (*n* = 10). Quantitative data are indicated as mean ± SEM. *p < 0.05; **p < 0.01; ***p < 0.001; ns indicates no significant (*P* > 0.05).

Vascular pericyte coverage is an important marker for vascular maturity [18, 19]. As vascular maturity is the basis for vascular function [20], we next focused on pericyte coverage by vascular smooth muscle cells (VSMCs).We performed 3D-reconstruction of immunofluorescent double-staining for CD31 and α-SMA (alpha smooth muscle actin), a specific marker for VSMC. In PNR, irregular vascular lumens were almost uncoated by VSMCs (Figure 3A), indicating a complete absence of pericyte coverage on vessels in PNR. Unlike tumor vessels, vessels distributed in normal breast tissue of BALB/c mouse were well coated by VSMCs (Figure 3B), suggesting a mature vasculature.

To study the effect of metformin on tumor vasculature, mice were orally administrated with metformin. Compared to untreated tumor (Figure 3A), metformin-treated tumor exhibited a significantly promoted vascular maturity (Figure 3C, 3D). The change was indicated by increased ratio of co-located α-SMA signal to CD31 signal in PNR, TR and NNR (Figure 3D). Furthermore, metformin apparently reduced tumor necrosis, MVD and vascular branch density in PNR (Figure 3E–3J), while having no effect on angiogenesis in both TR and NNR (Figure 3E–3J). Thus, metformin-induced reduction in average MVD and vascular branch density of whole tumor region mainly resulted from significantly inhibited angiogenesis in PNR.

### Metformin suppressed HIF-1α-induced AAFs in PNR

**Figure 4:**
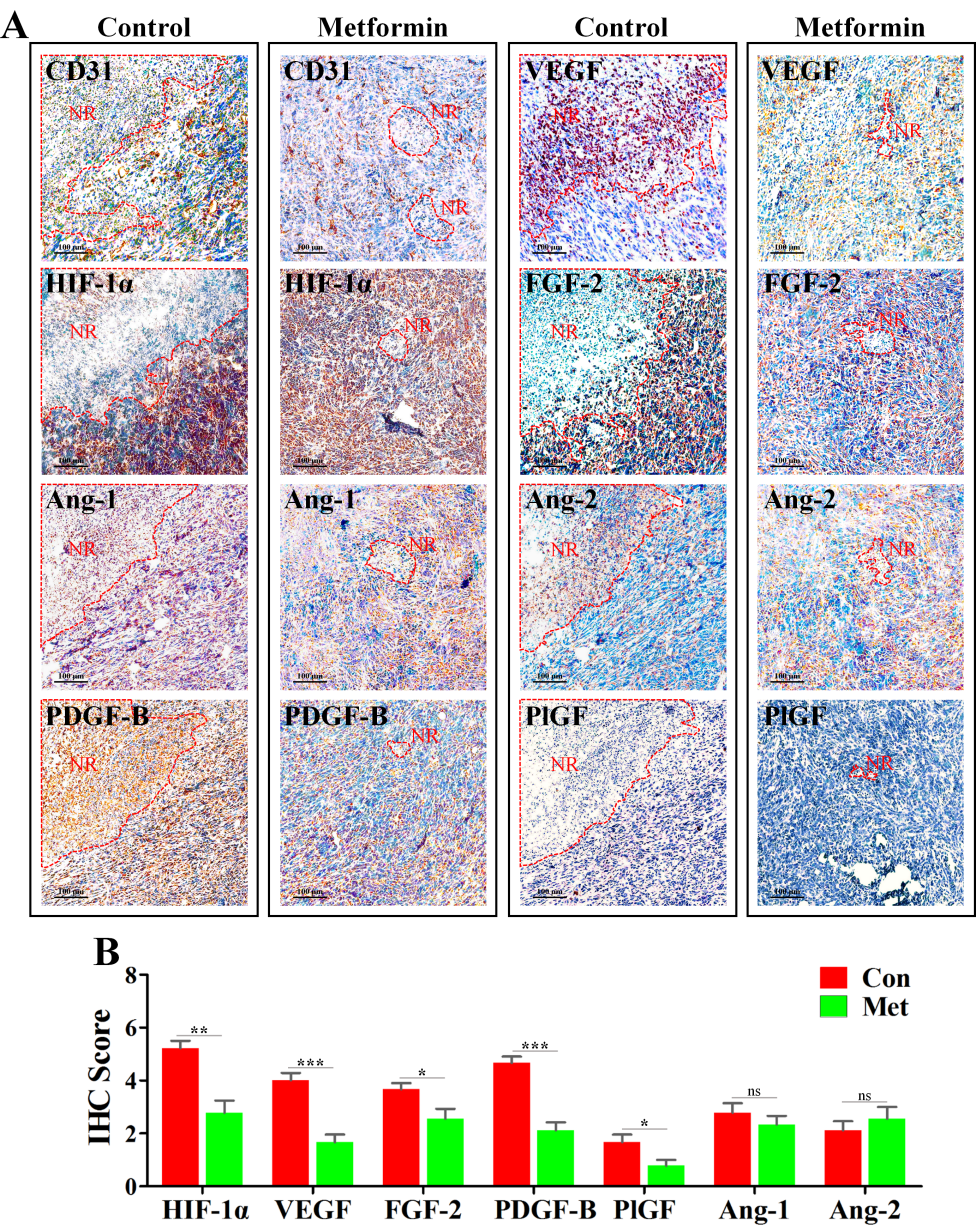
Metformin inhibited angiogenesis in peri-necrotic region by impeding HIF-1α-induced expressions of pro-angiogenic factors 4T1 tumors extracted from untreated or metformin-treated mice were embedded in paraffin and cut into 4–5 μm for IHC staining. (**A**) IHC staining for CD31, HIF-1α, Ang-1, Ang-2, PDGF-B, PlGF, VEGF and FGF-2 in untreated or metformin-treated BALB/c mice. Scale bar: 100 μm. Necrotic region is indicated by “NR” and its boundary is surrounded by red dotted line. (**B**) Quantification of IHC scores (addition of intensity score and positive signal area) of angiogenesis-associated factors, including HIF-1α, Ang-1, Ang-2, PDGF-B, PlGF, VEGF and FGF-2 (*n* = 10). Quantitative data are indicated as mean ± SEM. *p < 0.05; **p < 0.01; ***p < 0.001; ns indicates no significant (*P* > 0.05).

HIF-1α is one of the most critical factors for mediating angiogenesis in hypoxic condition [21, 22]. To explore the mechanism leading to inhibition of angiogenesis in PNR, we next investigated the change of HIF-1α and AAFs. Further characterization of IHC staining showed that HIF-1α, VEGF, fibroblast growth factor-2 (FGF-2) and platelet-derived growth factor-B (PDGF-B) were highly expressed in the edge region of necrosis (Figure 4A), while angiopoietin-1 (Ang-1), angiopoietin-2 (Ang-2) and placental growth factor (PlGF) exhibiting weaker expression level. Further, to determine whether metformin-induced inhibition of PNR angiogenesis resulted from down-regulated AAFs, we focused on the change of AAFs in PNR. As expected, expression levels of HIF-1αand AAFs, including VEGF, PDGF-B, FGF-2 and PlGF, were significantly reduced in PNR of metformin-treated 4T1 tumor tissue. (Figure 4B) However, both Ang-1 and Ang-2 were not affected by metformin administration.

### Tumor hypoxia was ameliorated by metformin via elevating tumor blood perfusion

**Figure 5:**
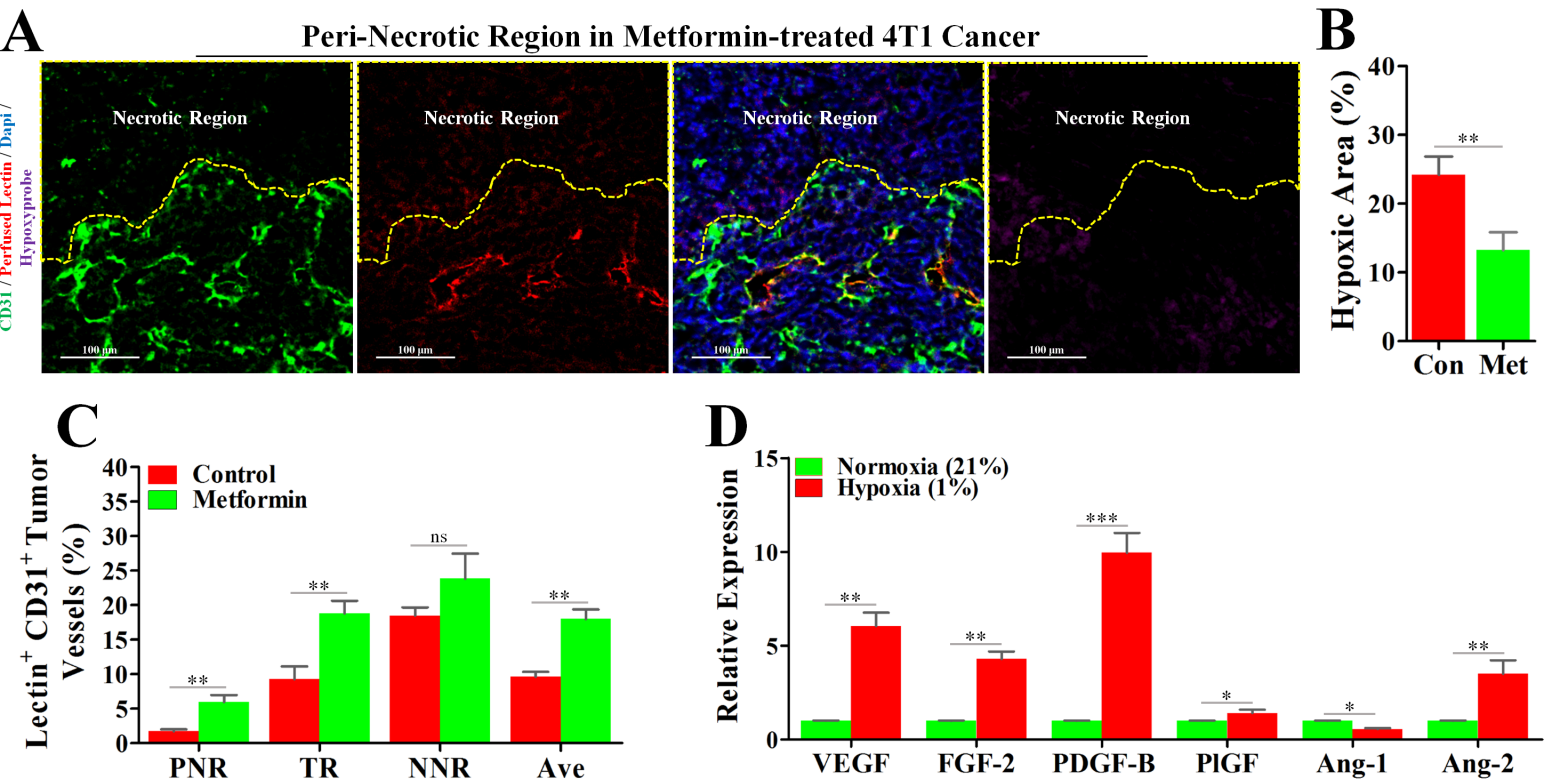
Metformin inhibited angiogenesis in peri-necrotic region by impeding HIF-1α-induced expressions of pro-angiogenic factors 4T1 tumor-bearing mice were untreated (con) or persistently treated metformin (1.5 mg/mL) for 21days. Before extraction, mice were sequentially injected with pimonidazole and TRITC-conjugated lectin. Staining for CD31 (Green), TRITC-conjugated lectin (Red) and pimonidazole hydrochloride (Violet) in the peri-necrotic region of 4T1 tumors from metformin-treated BALB/c mouse. The necrotic region was surrounded by yellow dotted line. Scale bar: 100 μm. (**B**) Quantification of percentage of hypoxic area (indicated by hypoxyprobe positive area) in 4T1 tumors from untreated or metformin-treated BALB/c mice (*n* = 10). (**C**) Percentage of perfused-lectin^+^ CD31^+^ vessels in all CD31^+^ vessels of the whole tumor area (Ave indicates average perfusion level), peri-necrotic region (PNR), transitional region (TR) and non-necrotic region (NNR, *n* = 10). Mice were untreated or persistently treated with metformin. (**D**) Increased relative mRNA expressions (to normoxia) of various angiogenic factors of 4T1 cancer cells cultured in the hypoxic condition (1% O^2^). mRNA level was detected by real-time quantitative PCR. Quantitative data are indicated as mean ± SEM. *p < 0.05; **p < 0.01; ***p < 0.001; ns indicates no significant (*P* > 0.05).

In light of the reports that metformin has the potential to improved tumor oxygenation [13], we considered the possibility that metformin-induced inhibition of hypoxic angiogenesis might operate via increase of blood perfusion. Interesting, the unperfused condition of vessels in PNR was apparently reversed by a continuous administration of metformin (Figure 1C and Figure 5A), which was consistently accompanied by a significant reduction in hypoxia (Figure 5B). Further characterization of tumor blood perfusion showed that metformin increased average perfusion via elevation of perfusion in both TR and PNR (Figure 5C).

### Normoxia abrogated hypoxia-induced expressions of pro-angiogenic factors of 4T1 cancer cell *in vitro*

To simulate the *in vivo* tumor hypoxia, 4T1 cancer cells were cultured under 21% O^2^ and 1% O^2^, respectively, and then mRNA levels of AAFs were detected by real-time quantitative PCR. As shown in Figure 5D, high mRNA expression levels of pro-angiogenic factors, including VEGF, FGF-2, PDGF-B, PlGF and Ang-2, in 4T1 cells cultured in hypoxic condition were significantly reduced by normoxia. Unsimilarly to pro-angiogenic factors, Ang-1, an anti-angiogenic factor, exhibited an increased mRNA level in normoxic condition. Thus, it appears that increased blood perfusion is able to ameliorate hypoxia-induced expressions of pro-angiogenic factors.

### Knock-down of HIF-1α abrogated hypoxia-induced angiogenesis and reduced vascular leakage

**Figure 6:**
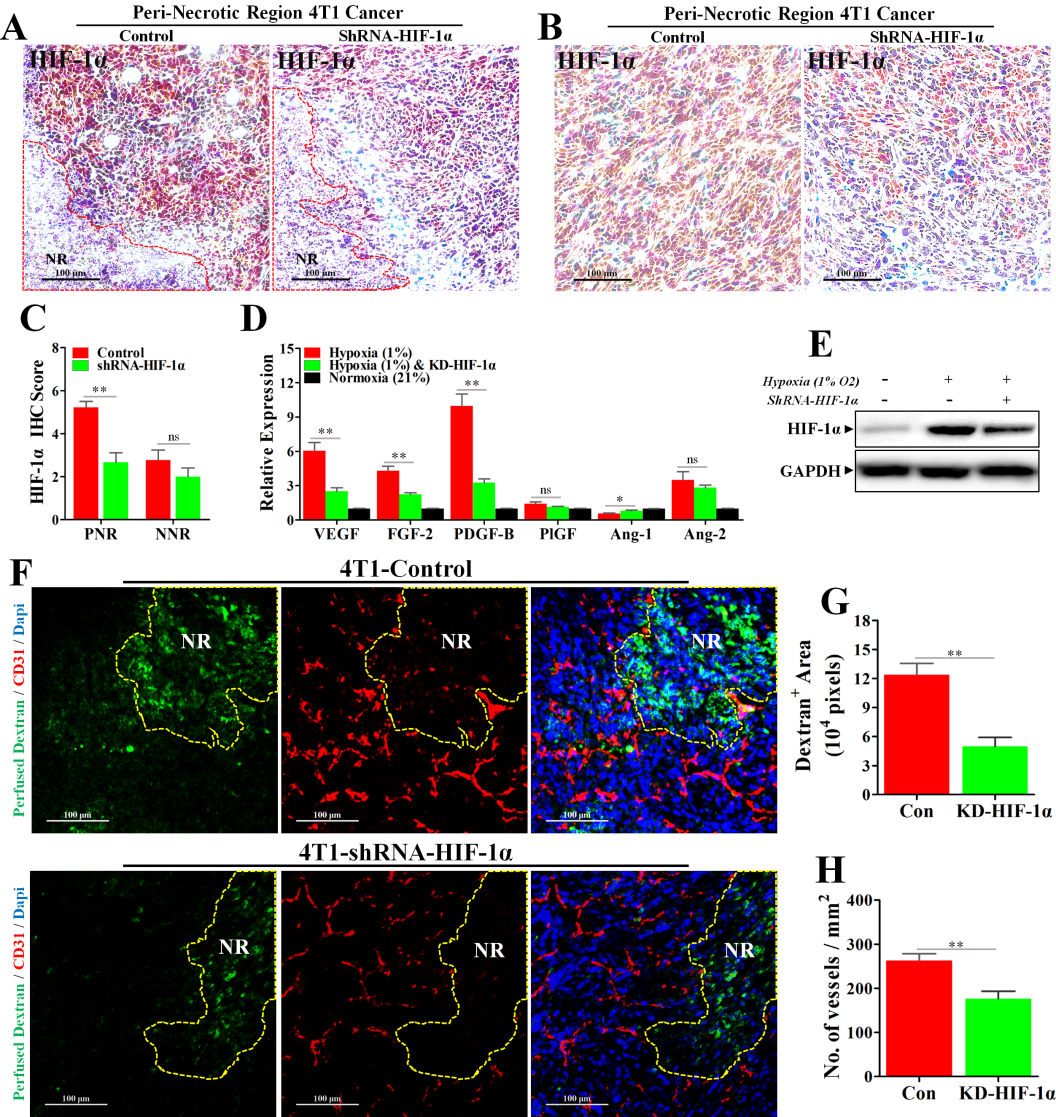
Knock-down of HIF-1α inhibited hypoxia-induced abnormal angiogenesis and reduced vessel leakage 4T1 cancer cells transfected with Lentivirus-shRNA-HIF-1α or Lentivirus-shRNA-Con were orthotopically transplanted into the BALB/c mice. (**A**–**C**) IHC staining for HIF-1α, revealing a significant reduction of HIF-1α protein level in both (A) peri-necrotic and (B) non-necrotic regions of shRNA-HIF-1α 4T1 tumors; (C) quantification of HIF-1α IHC score (addition of intensity score and positive signal area). *n* = 9; scale bar: 100 μm. (**D**) Reduced relative mRNA expressions of various angiogenesis-associated factors in shRNA-HIF-1α 4T1 cancer cells cultured in the hypoxic condition (1% O^2^). *n* = 6. (**E**) Immunoblotting for HIF-1α, showing reduced HIF-1α protein level in Lentivirus-shRNA-HIF-1α 4T1 cancer cells cultured in hypoxic condition (1% O^2^) *in vitro*. 100 μg cellular protein was loaded on each lane. (**F**) Staining for fitc-conjugated dextran (green, 70 kD) and CD31 (red) in 4T1 tumors transfected with Lentivirus-shRNA-HIF-1α or Lentivirus-shRNA-Con. Before extraction, fitc-conjugated dextran was intravenously injected into the tumor-bearing mice for observation of vascular leakage. NR indicates necrotic tumor region. Scale bar: 100 μm. (**G** and **H**) Quantification of (G) detran^+^ area leaking outside the tumor lumen and (H) microvessel density (No. of vessels per mm^2^) in 4T1 tumors transfected with either Lentivirus-shRNA-HIF-1α or Lentivirus-shRNA-Con (*n* = 10). Quantitative data are indicated as mean ± SEM. *p < 0.05; **p < 0.01; ***p < 0.001; ns indicates no significant (*P* > 0.05).

To further delineate the role of HIF-1α in mediating hypoxia-induced angiogenesis, we established the 4T1 cell line with stable HIF-1α knock-down. The knock-down efficiency was first verified *in vivo*. Our results of IHC staining for HIF-1α (Figure 6A–6C) showed that HIF-1α expression was significantly inhibited in PNR, while HIF-1α of NNR exhibiting slight reduction. These data indicated a successful knock-down of HIF-1α *in vivo*. Furthermore, knock-down of HIF-1α also significantly abrogated hypoxia-induced expressions of AAFs of 4T1 cells both *in vitro* and *in vivo* (Figure 6D, 6E and Supplementary Figure 1D, 1E).

**Figure 7:**
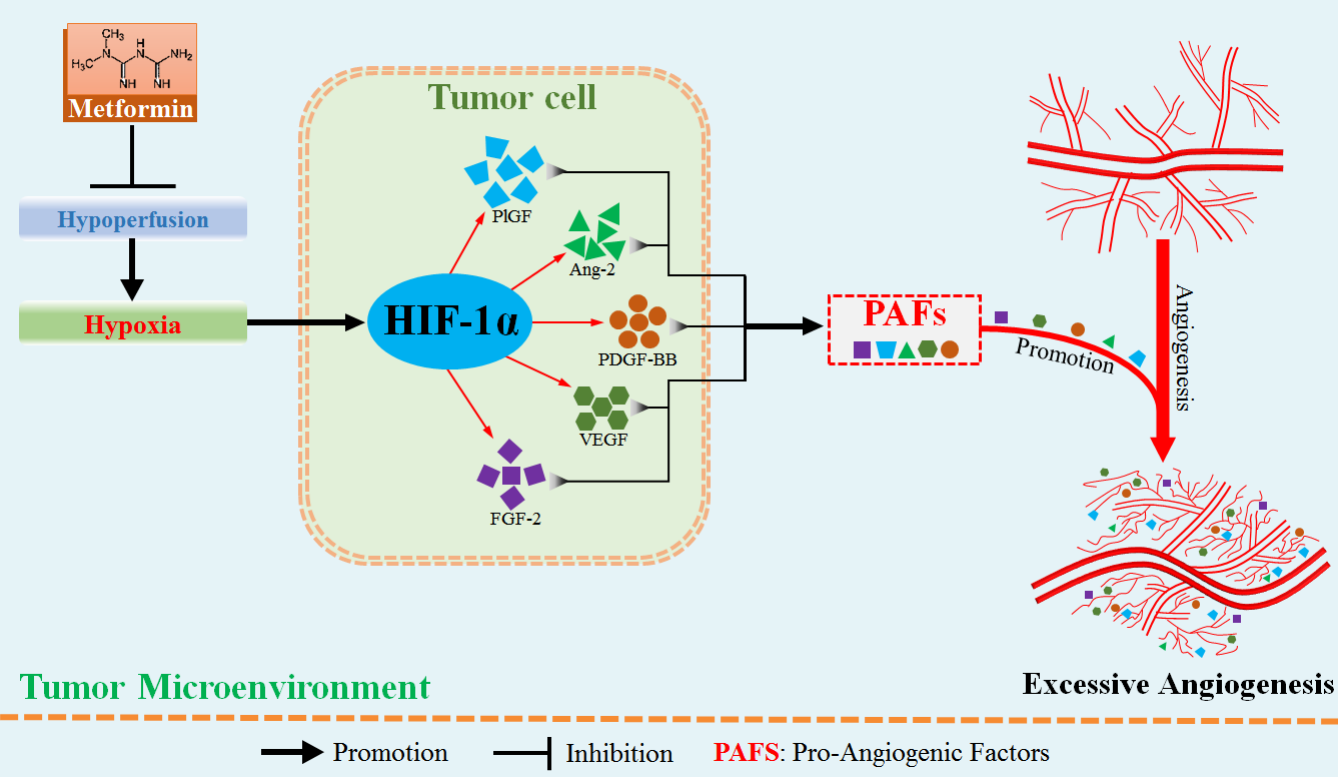
Metformin administration suppressed hypoxia-induced abnormally excessive angiogenesis by ameliorating tumor hypoperfusion In the hypoxic region adjacent to tumor necrosis, hypoperfusion results in aberrant angiogenesis via hypoxia-induced increase in HIF-1α protein level. HIF-1α protein has the potential to up-regulate multiple pro-angiogenic factors, thus greatly enhancing the abilities of sprouting and branching of tumor vessels. Metformin suppresses the abnormally aberrant angiogenesis in tumor by inhibiting HIF-1α-induced expressions of pro-angigenic factors via increase of tumor perfusion.

Given aberrant tumor vessel is characterized by intense vascular leakage, we next investigated whether blockade of HIF-1α would reduce the vascular leakage, which was indicated by fitc-conjugated dextran leaking outside the vascular lumen. We found that dextran^+^ area distributed outside tumor vessel was significantly reduced by 7.32 × 10^4^ pixels in shRNA-HIF-1α-4T1 tumor, suggesting an inhibitory effect of HIF-1α blockade on vessel leakage. (Figure 6F–6G) Moreover, vessels of shRNA-HIF-1α-4T1 tumor exhibited apparently weaker ability of angiogenesis in PNR than shRNA-control-4T1 tumor. (Figure 6F and 6H) Taken together, these results show that hypoxia-induced HIF-1α promotes aberrant angiogenesis in PNR by up-regulation of pro-angiogenic factors. Amelioration of tumor hypoxia by metformin treatment suppresses excessive angiogenesis via inhibition of HIF-1α-AAFs signaling. The associated mechanism responsible for metformin-induced inhibition of excessive angiogenesis is illustrated in Figure 7.

## DISCUSSION

Herein, we report that metformin administration significantly inhibits excessive angiogenesis in murine breast cancer model. Support this conclusion comes from our observations that tumor angiogenesis resulted from hypoxia and was apparently inhibited by metformin via elevating tumor blood perfusion. Critically, metformin-induced amelioration of hypoxia led to inhibited expressions of AAFs and consequently restrained angiogenesis in murine breast 4T1 carcinoma. Thus, unlike the direct drug activities reported in previously published articles [7, 23], in our study, hypoxic microenvironment was greatly contributed to the excessive and dysregulated angiogenesis, and we show that this was mediated by HIF-1α-induced expressions of AAFs, especially the pro-angiogenic factors.

Our results are consistent with the previous pre-clinical report of metformin-mediated amelioration of tumor hypoxia [13], and provided complementary information about why tumor oxygenation was elevated. By elevating tumor blood perfusion, more oxygenation is delivered to tumor region, especially the deep region, and lead to a corresponding reduction in tumor hypoxia. This result may reflect the greater dependence of abnormal tumor angiogenesis on hypoxia. Hypoxic tumor microenvironment correlates with advanced stage of the malignancies and greatly contributes to tumor resistance to anti-angiogenic, immune and chemical therapies [2, 24, 25]. Based on these evidences, reprogramming hypoxic tumor microenvironment has been widely considered as a promising therapeutic strategy [26, 27]. Thus, the therapeutic significance of metformin-induced amelioration of hypoxia should not be simply limited to inhibition of tumor angiogenesis.

Our results show that metformin is similar with the typical vascular-targeting agents in increasing blood perfusion [28], but dissimilar with the vascular-promoting therapy [29]. Although both therapies have similar effects on elevating blood perfusion, their influence on tumor vasculature is completely different. Vessels in tumors treated with vascular promoting agents were reported to be dilated and with leakage [30]. Our results showed that metformin-treated tumor exhibited a more mature vasculature, which was indicated by increased vascular pericyte coverage in peri-necrotic hypoxic region. Since a mature vasculature is the fundamental basis for efficient blood transportation, this result may give explanation to the elevated blood perfusion. Taken together, our data warrant further studies to elucidate the mechanism underlying the effects of metformin on promoting tumor vascular maturity.

Previous studies were mainly focused on the direct effects of metformin on tumor cells or endothelial cells [23, 31]. A growing number of studies started to confirm that metformin could benefit patients via indirect mechanisms [10, 32, 33]. Our results provide a novel insight into the indirect mechanistic profile leading to inhibited angiogenesis and ameliorated microenvironment by metformin. Additionally, inhibition of AAFs, including VEGF, FGF-2, PDGF-B and PlGF, expands our understanding of angiogenic suppression by metformin, because previous article mainly concentrated in the involvement of the downstream VEGF [10]. These changes of AAFs are closely associated with reduced HIF-1α protein level, which results from ameliorated hypoxia. Moreover, metformin was reported to directly reduce HIF-1α protein level in normoxia [10, 34]. This is further supported by the fact that metformin decreased angiogenic ability of vessels even in non-hypoxia region.

Our results indicate that metformin can substantially inhibit tumor angiogenesis by ameliorating tumor hypoxia that has potential therapeutic implications. First, according to the knowledge of tumor response to radiotherapy, metformin-induced amelioration of tumor hypoxia would increase tumor sensitivity to radiotherapy [13, 35]. Second, the reprogramed hypoxic microenvironment would benefit patients due to reduced therapeutic resistances to chemo-drug, immune therapy and anti-angiogenic treatment [10, 32, 36, 37]. Third, novel strategies to overcome the barrier of hypoxia in clinical treatment are in dire need, and the anti-diabetic metformin may represent as a workable and safe approach. Fourth, biomarker should be incorporated to predict for patient response to metformin pretreatment. Fifth, angiogenesis-dependent tumors are responsive to metformin, but less has been known about angiogenesis-independent tumors.

In summary, hypoxia-mediated aberrant and excessive angiogenesis was significantly inhibited by metformin administration, which provides a novel rationale for metformin as a new treatment for malignant tumors.

## MATERIALS AND METHODS

### Cells

Mouse breast carcinoma 4T1 cell was obtained from the American Type Culture Collection (ATCC, Manassas, VA) and cultured with Dulbecco’s modified Eagle’s medium (DMEM, Invitrogen) supplemented with 10% fetal bovine serum (FBS, Invitrogen) in an atmosphere of 5% CO^2^ and 95% room air at 37°C. Hypoxic culture condition was realized by using a hypoxia incubator chamber that could reduce the O^2^ to 1%.

### Generation of HIF-1α knock-down cells

Mouse lentiviral shRNA-HIF-1α and vector shRNA constructs were purchased from SantaCruz (sc-35562-V; sc-108080). Vector shRNA lentiviral particle containing scrambled shRNA sequence was used as a negative control. 4T1 cells were transduced with in a mixture of complete medium and Polybrene (Sigma) at a final concentration of 8 μg/ml. Transduced cells were finally selected by 2.5 μg/ml puromycin (Cayman). Serial dilution and HIF-1α expression screen by western blot were performed under 250 μM CoCl_2_ (Sigma) treatment.

### Animal models

All animal experimental protocols were approved by the Ethical Committee and the Institutional Animal Care and Use Committee of Xi’an Jiaotong University. Briefly, 1 × 10^6^ untransfected 4T1 cells or 4T1 cells with either lentiviral shRNA-HIF-1α or shRNA-Control was injected into the fat pad of 4th breast of 6–8 weeks old female BALB/C mice for establishment of an orthotopical breast cancer model. For metformin administration, metformin was advancedly added to drinking water [10]. 28 days after inoculation, mice were sacrificed by abdominal injection of 20% ethylcarbamate and the whole tumor tissue was extracted.

### Immunoblotting

Protein concentration was determined by Protein Assay Kit (Bio-Rad). For detection of HIF-1α, 100 μg proteins were loaded on each lane and separated by 10% SDS-PAGE. Antibodies for HIF-1α (Abcam), VEGF (ProteinTech) and FGF-2 (Sangon Biotech) were used at a dilution of 1:750, 1:2000 and 1: 4000, respectively, and followed by HRP-conjugated secondary antibody (ProteinTech). A chemiluminescence imaging system was used for quantification of protein expression (Bio-Rad).

### Real-time PCR

To detect mRNA levels of AAFs, a standardized real-time PCR was performed by using SYBR green Master Mix (Takara) according the manufacturer’s instructions. All PCR primers used in the present study were listed in Supplementary Table 1.

### Vascular leakage, perfusion and tumor hypoxia

For observation of vascular leakage, 100 μl fitc-conjugated dextran (100 mg/ml, 70 kDa; Sigma) was injected into the tail vein of BALB/c mice 30 min before sacrifice. Tumors were fixed in 4% paraformaldehyde (PFA) for 8 h, immersed in 30% sucrose, embedded in OCT and finally cut into 6μm sections. The green fitc signal outside the vascular wall (stained with PE-conjugated CD31 antibody, Biolegend) was considered as the leaked dextran. For determination of vascular perfusion and tumor hypoxia in the same area, pimonidazole (PIMO, Cayman) and TRITC-conjugated lectin (Vector Lab) were intravenously injected into the same mouse 2 h and 15 min before extraction, respectively. The advancedly prepared tumor sections were then incubated with the mixture of APC-conjugated anti-PIMO antibody (Hypoxyprobe), fitc-conjugated anti-CD31 antibody and 5% BSA overnight.

### Immunofluorescence and immunohistochemistry

For immunohistochemistry, tumor tissues were fixed in 10% formaldehyde solution, embedded with paraffin and cut into 4–5 μm thick sections. A standard immunohistochemistry staining method was applied for observation of the change of expressions of angiogenesis-associated factors in peri-necrotic region [15]. All antibody applied could be found in Supplementary Table 1. The result of whole section was finally recorded by using a slide-scanner (Leica). Tumor regions were divided to NR, PNR, TR and NNR according to the histological appearance. NR is the avascular tumor region, where tissue cells are engaged in nuclear condensation and fragmentation. PNR is the excessively vascularized region adjacent to NR, where the average vascular branches per vessels is always higher than 5, whilst that of NNR is less than 3. TR locates between PNR and NNR.

To observe vascular pericyte coverage, 4% PFA-fixed tumor tissue was cut into 40 μm sections. Sections were incubated with the mixture of anti-CD31 antibody (Abcam) and anti-α-SMA antibody (Boster) for 24 h at 4°C, followed by staining with appropriate fluorescently conjugated secondary antibodies (Invitrogen). Nuclei were counterstained by DAPI (5 µg/mL) for 15 min at room temperature before imaging. Images of multi-layers (4–5 µm per layer) were recorded by using a confocal laser scanning microscopy (Leica). 3D-reconstrution of CD31/α-SMA fluorescent signaling was performed by using the software of LAS AF Lite (Leica).

### Statistical analysis

Quantitative data were represented as mean ± SEM. Two-way ANOVA, one-way ANOVA and Student’s *t* test were used to perform statistical test. Spearman analysis was performed for determination of the linear correlation between MVD of different tumor regions or MVD and necrosis. *P < 0.05, **P < 0.01, ***P < 0.001; ns indicates no significance.

## SUPPLEMENTARY MATERIALS FIGURE AND TABLE



## Abbreviations

α-SMA: alpha smooth muscle actin
AAFs: angiogenesis-associated factors
Ang-1: angiopoietin-1
Ang-2: angiopoietin-2
ATCC: American Type Culture Collection
DMEM: dulbecco modified eagle medium
FBS: fetal bovine serum
FGF-2: fibroblast growth factor-2
HER2: human epidermal receptor 2
HIF-1α: hypoxia inducible factor 1 alpha
HRP: horseradish peroxidase
MVD: microvessel density
NNR: non-necrotic region
PDGF-B: platelet-derived growth factor-B
PIMO: pimonidazole
PlGF: placental growth factor
PNR: peri-necrotic region
SDS-PAGE: sodium dodecyl sulfate polyacrylamide gel electrophoresis
shRNA: small hairpin RNA
TR: transitional region
VSMCs: vascular smooth muscle cells
TRITC: tetramethyl rhodamin isothiocyanate
VEGF: vascular endothelial growth factor
