# Supplementary material for: Suppression of hypoxia-induced excessive angiogenesis by metformin via elevating tumor blood perfusion
Source: Oncotarget. 2017 May 19;8(43):73892–904. doi: 10.18632/oncotarget.18029 (PMC5650310; doi:10.18632/oncotarget.18029)
Supplement: Supplementary file 1 [file oncotarget-08-73892-s001.pdf]

# Suppression of hypoxia-induced excessive angiogenesis by metformin via elevating tumor blood perfusion

## SUPPLEMENTARY MATERIALS

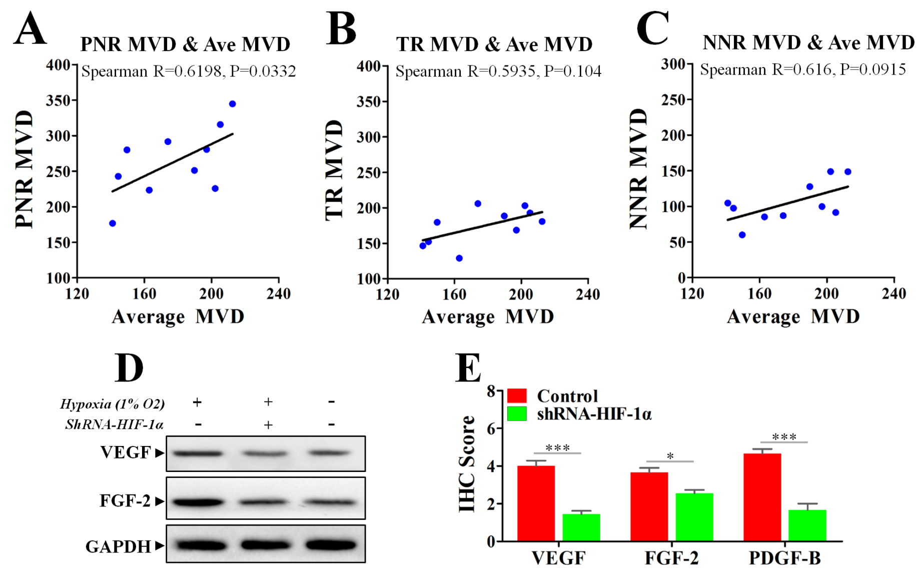


**Supplementary Figure 1: PNR but not NNR or TR MVD was linearly correlated with the Ave MVD, and knockdown of HIF-1α restrained hypoxia-induced expression of angiogenesis-associated factors.** Spearman analysis for evaluation of the linear correlation between Ave MVD and (**A**) PNR, (**B**) TR or (**C**) NNR MVD. Spearman R indicates the degree of correlation. Ave: average; MVD: microvessel density; NNR: non-necrotic region; PNR: peri-necrotic region; TR: transitional region. A two-sided $P < 0.05$ was considered statistically significant. (**D**) Immunoblotting for VEGF, FGF-2 and PDGF-B, showing reduced protein level of angiogenesis-associated factors in Lentivirus-shRNA-HIF-1α 4T1 cancer cells cultured in hypoxic condition (1% $O^2$) *in vitro*. (**E**) Quantification of VEGF, FGF-2 and PDGF-B IHC score (addition of intensity score and positive signal area) in 4T1 tumors transfected with Lentivirus-shRNA-HIF-1α or Lentivirus-shRNA-Con. $n = 9$. Quantitative data are indicated as mean ± SEM. *$p < 0.05$; **$p < 0.01$; ***$p < 0.001$.

**Supplementary Table 1: Primers and antibodies**

**Primers**

| Gene | Forward Primer | Reverse Primer |
|---|---|---|
| Act-β | GACATCCGTAAAGACCTCTATGCC | TCCACACAGAGTACTTGCGCTCA |
| Ang-1 | CAAATGCGCTCTCATGCTA | GTAGTGCCACTTTATCCCAT |
| Ang-2 | CGCTGTATGATCACTTCTACCTC | TCCTGAGAGCATCTGGGAAC |
| FGF-2 | GGCTGCTGGCTTCTAAGTGT | TCTGTCCAGGTCCCGTTTTG |
| HIF-1α | AGTGGTATTATTCAGCACGAC | AAGGCAGCTTGTATCCTCT |
| PDGF-B | TCTCTGCTGCTACCTGCGTCT | CAGCCCCATCTTCATCTACGG |
| PlGF | GTCTCACATATTCAGTCCGTCC | TTGGCTGTCTTTATCGGCAC |
| VEGF | GTGCAGGCTGCTGTAACGAT | GGGATTTCTTGCGCTTTCGT |

**Antibodies for IHC**

| Antibody | Company, Species | Concentration |
|---|---|---|
| Anti-CD31 | Abcam, Rabbit | 1 μg/ml |
| Anti-HIF-1α | Abcam, Rabbit | 1.5 μg/ml |
| Anti-PlGF | ProteinTech, Rabbit | 1.5 μg/ml |
| Anti-PDGF-B | Boster, Rabbit | 1.5 μg/ml |
| Anti-Ang-1 | Boster, Rabbit | 1.5 μg/ml |
| Anti-Ang-2 | Boster, Rabbit | 1.5 μg/ml |
| Anti-VEGF | ProteinTech, Rabbit | 1.5 μg/ml |
| Anti-FGF-2 | ProteinTech, Rabbit | 1.5 μg/ml |
